# Review of developments in corneal transplantation in the regions of Brazil - Evaluation of corneal transplants in Brazil

**DOI:** 10.6061/clinics/2016(09)09

**Published:** 2016-09

**Authors:** Hirlana Gomes Almeida, Richard Yudi Hida, Newton Kara-Junior

**Affiliations:** I Hospital das Clínicas da Faculdade de Medicina da Universidade de São Paulo, Departamento de Oftalmologia, São Paulo/SP, Brazil; II Santa Casa de São Paulo, Departamento de Oftalmologia, São Paulo/SP, Brazil

**Keywords:** Ophthalmology, Health Profile, Diagnosis, Corneal Diseases, Corneal Transplantation

## Abstract

The aim of this study was to identify inequalities in corneal donation and transplantation among the regions of Brazil. A transversal and retrospective study was specifically conducted using data from the Brazilian Transplant Registry collected by the Brazilian Association of Organ Transplantation between January 2002 and December 2014. The collected data were processed using descriptive statistical methods, and *p*<0.05 was the rate of rejection of the null hypothesis. From 2002 to 2014, there was an increase in the absolute number of corneal transplants, the annual rate of transplants per million people and the percentage of needed transplants performed in each of the five regions of Brazil. Family refusal and medical contraindication were the most frequent reasons for a lack of corneal donation. Although remarkable progress has been made in the last decade in each of the five Brazilian regions, health professionals’ lack of preparation to approach families with donation requests at the death of a family member appears to be the main obstacle to increasing the number of corneal donations. Thus, the present study suggests the implementation of public policies to make corneal transplants more effective, particularly given that there are considerable disparities in the effectiveness with which regional needs are met and in health professionals’ ability to perform transplants among the Brazilian regions, with higher rates in the South, Southeast and Midwest regions and lower rates in the North and Northeast regions.

## INTRODUCTION

In Brazil, the number of corneal transplants increased from 4,976 in 2002 to 13,036 in 2014 1. However, there continue to be major disparities in the ability to perform the surgery, the number of surgeries performed, and the effectiveness with which transplant needs are met across the regions of Brazil 2.

The major obstacle is the number of donated corneas, which, despite a progressive increase, is less than the number of potential donors and does not meet the requirements of the population 3.

The Brazilian Constitution establishes that health is the right of all and a duty of the state, which should ensure equitable universal access to medical procedures and health services 4. However, though organ and tissue transplants are a fundamental breakthrough in medicine, equal access to transplants has not yet been achieved in every Brazilian region. These inequalities compromise the fairness of the provision of health services to the population 5.

In Brazil, the heterogeneity of the regions is justified in part by geographical factors. However, few studies in the literature have shown differences in indicators and other relevant variables related to organ and tissue transplantation among the five Brazilian regions.

Assessing the health of a population using indicators provides valuable information for the development of social policies related to organ transplantation that can help governments and societies to promote health and social welfare 5.

The purpose of the present study was to identify inequalities in corneal donation and transplantation among the regions of Brazil.

## MATERIALS AND METHODS

This study adhered to the tenets of the Declaration of Helsinki and was approved by the Institutional Review Board/Ethics Committee of the Hospital das Clínicas da Faculdade de Medicina da Universidade de São Paulo (protocol number 099/14, session 04/16/2014).

A descriptive, retrospective database review was performed by an author of this article (H.G.A.) during February and March 2015. The records of 141,270 corneal transplantations performed between January 2002 and December 2014 were specifically retrieved from the computer database of the Brazilian Transplant Registry (RBT). All data were obtained from official documents of the Brazilian Organ Transplant Association (ABTO) (www.abto.org.br).

The following variables were analyzed: (1) the absolute number of individuals on the corneal transplant waiting list in each Brazilian region from 2012 to 2014 (data for previous years were not available), (2) the absolute number of corneal transplantations performed per year (T/Y) in each of the five Brazilian regions, (3) the absolute number of corneal transplantations pmp per year (T/mi/Y) in each Brazilian region, and (4) the reasons for the inability to perform organ and tissue donations per year (IOD/Y) in each of the five Brazilian regions.

The states studied were categorized into the following regions: North, Northeast, Midwest, Southeast and South. These regions are shown on the geographical map of Brazil in Appendix 1.

The absolute number of individuals on the corneal transplant waiting list in December of each year from 2012 to 2014 in each Brazilian region is cited to illustrate the heterogeneity of the population’s access to corneal transplantation across the regions.

The T/mi/Y in each region indicated the effectiveness with which the needs of the population were met. This value was not calculated in the current study but was rather based on the data provided by official documents of the RBT.

The demand for corneal transplantation in 2012, 2013 and 2014 (DCT/Y) was calculated by Brazilian region based on the following formula: DCT/Y=CT/CT+WL.

CT=the number of corneal transplantations performed in the region in each year.

WL=the number of persons on the waiting list the following year in the region.

This indicator (DCT/Y) was calculated to quantify what percentage of needed transplants were actually performed in each region each year. Data from the previous year’s waiting list, required to calculate the DCT/Y, were not available in the RBT.

The reasons for the inability to perform organ donation per year (IOD/Y) from 2002 to 2014 in each Brazilian region were divided into five categories: NAF (family refusal), CIM (medical contraindications), BDNC (brain death not confirmed), II (inadequate infrastructure) and other (the RBT does not explain the causes included in this category).

Certain data were not available in the RBT, as indicated in the tables.

## RESULTS

Table 1 shows the absolute number of individuals on the waiting list in December of each year from 2002 to 2014 in each of the regions. Data for previous years were not available in the RBT.

Table 2 shows the demand for corneal transplantation per year (DCT/Y) from 2012 to 2014 in each Brazilian region. The Midwest, Southeast and South regions met more than 80% of the need for corneal transplantation in 2013. The RBT did not provide data on the number of patients on the corneal transplant waiting list from 2002 to 2011, and it was therefore impossible to calculate the DCT/Y for these years.

Table 3 shows the reasons for the inability to perform organ donation per year (IOD/Y) by Brazilian region and the absolute number of cases of each reason from 2002 to 2014. Notably, the RBT data did not differentiate between refusal to donate organs and refusal to donate tissues. Thus, the authors assumed that the refusal data applied to both organs and tissues, including corneal donation refusal.

**Figure 1:**
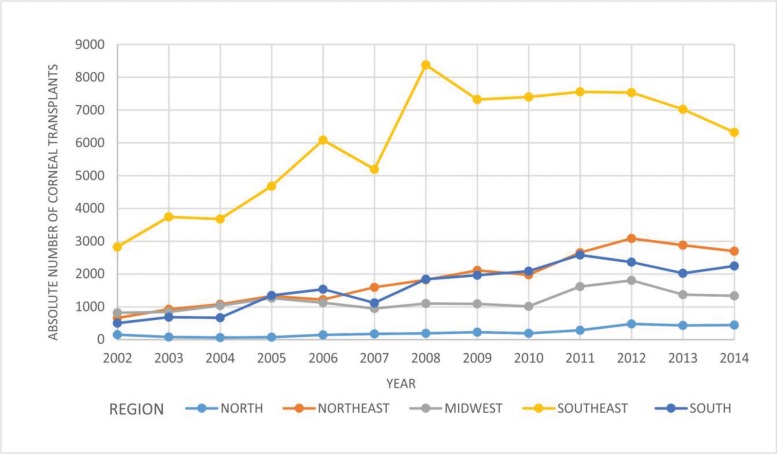
Absolute number of annual corneal transplants in each Brazilian region from 2002 to 2014.

Figure 1 shows the absolute number of annual corneal transplants performed in each of the five Brazilian regions from 2002 to 2014. In the Northeast and South regions, the absolute number of transplants performed between 2002 and 2014 quadrupled.

**Figure 2:**
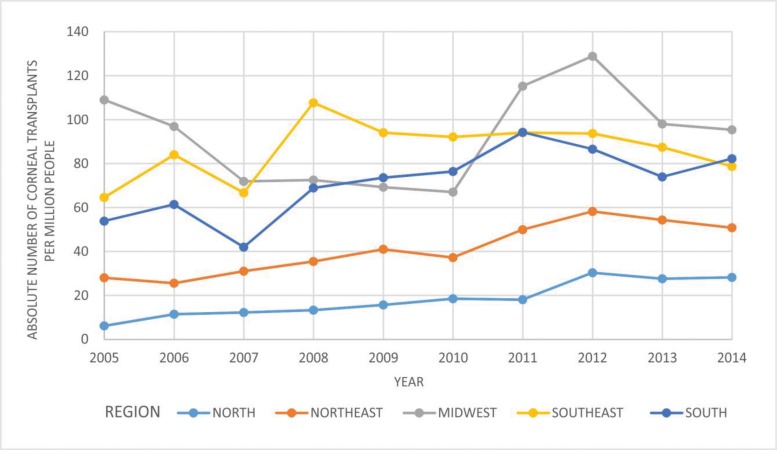
Absolute number of corneal transplants per million people in each Brazilian region from 2005 to 2014. Data from the years 2002, 2003 and 2004 were not available in the Brazilian Transplant Registry (RBT).

Figure 2 shows the effectiveness (T/mi/Y) in each of the five Brazilian regions from 2005 to 2014. Effectiveness data (T/mi/Y) for the years 2002, 2003 and 2004 were not available in the RBT.

## DISCUSSION

Corneal diseases are the second leading cause of reversible blindness in the world and result in both loss of visual capability and biopsychosocial damage 6.

Brazil is second in the world in terms of the absolute number of corneal transplants 2. However, in terms of the absolute number of corneal transplants pmp, Brazil ranks 21^st^ 2.

In 2001, the Ministry of Health established the National Program for the Implementation of Tissue Eye Banks, whose purpose was to increase the collection of corneas, to shorten the time recipients spend on the waiting list and to significantly increase the number of corneal transplants performed 2.

GM Ordinance n° 1.752 (2005) also established that all hospitals with more than 80 beds must have a Donation of Organs and Tissues for Transplantation Commission 7, but fewer than 10% of Brazilian hospitals have active teams 2.

The ABTO, founded in 1987, is a non-profit organization that aims to encourage organ donation; to provide technical and logistical support for the organization and operation of a centralized system for the notification, collection and distribution of tissues and organs; to stimulate research and collaborate on the dissemination of knowledge about organ and tissue transplantation; and to educate the population about the humanitarian, scientific and moral significance of organ donation for transplantation.

Each quarter, the ABTO publishes the RBT, a dynamic epidemiological profile of national transplant activity. The purpose of the RBT is to manage the results of transplant activities and to correlate these results with the constant changes observed in the legal provisions governing such activities.

The data released in the RBT are collected via an online system in which all of the teams able to perform transplants in Brazil are registered. These teams have an annual schedule for sending information. The data provided by the teams are compared to the statistics provided to the ABTO by the State Central (an official organ of the Ministry of Health), and only after this comparison and correction of any discrepancies is the final RBT file released to the states, transplantation teams and the general population.

The RBT data show that the regions with the greatest numbers of patients on the corneal transplant waiting list from 2012 to 2014 were the Northeast, the North and the Southeast, while the South had the lowest number (Table 1).

This finding is reinforced by Table 2, which shows that the more developed regions (the Southeast, South and Midwest) met more than 70% of the demand for corneal transplantation in 2012 and 2013. In contrast, in 2014, the North met only 27.5% and the Northeast, 47.2%.

Thus, the higher the percentage of the demand for corneal transplantation met per year was, the lower the waiting time for a transplant was. A study by Sobrinho et al. 8 found that between 2001 and 2009, most patients waited one to three years for a penetrating keratoplasty in the state of Pará (northern Brazil), but Almeida and Souza 9 observed that patients waited for one to six months in the state of Pernambuco (Northeast of Brazil).

These findings reflect the fact that the North and Northeast regions of Brazil still face many difficulties in the corneal capitation and donation processes. These problems are the result of young and unstructured programs, low reporting rates among potential donors, employee strikes, a lack of trained professionals and a lack of infrastructure at many hospitals 2.

In contrast, the Southeast, South and Center-West regions have the most effective programs for organ and tissue transplantation in Brazil. In fact, the state of Pernambuco cleared its waiting list in 2013 and 2014 and sent the surplus corneas processed to other states 2.

For each of the five Brazilian regions from 2002 to 2014, Figure 1 shows the absolute number of corneal transplants and Figure 2 shows the absolute number of corneal transplants pmp (an indicator of a region’s effectiveness in meeting the needs of the population). In all regions, these numbers increased.

In 2014, the absolute number of corneal transplants pmp in the states of Goiás, Santa Catarina, São Paulo and the Federal District were higher than the estimated need in Brazil (90 pmp for each state) 1. However, six states had fewer than 40 transplants pmp: four in the Northeast (Paraíba, Bahia, Maranhão and Alagoas), three in the North (Pará, Acre and Rondônia), one in the Southeast (Rio de Janeiro) and one in the Midwest (Mato Grosso) 1.

Importantly, three Brazilian states located in the North region (Amapá, Roraima and Tocantins) performed no corneal transplants in 2014 1.

These findings suggest unequal access to corneal transplantation among the Brazilian states, which may be the result of various factors, including the less developed operational capability of central Brazil, the heterogeneous distribution of transplantation teams and the difficulty of accessing treatment for low-income populations located in less developed regions 10.

Thus, the greatest numbers of corneal transplants are concentrated in the Midwest, Southeast and South, where programs are better structured and more established, receive support from the local government and have better prepared medical teams, all of which contribute to improving the health of the population 1.

One successful Brazilian example of correct implementation and effective performance is the “Banco de Olhos de Sorocaba-São Paulo”, which, through “responsible marketing”, undertook intensive campaigns to educate people about organ and tissue donation and encouraged an effective, active team approach. This organization achieved an increase of 968% in the number of donations over 20 years 11.

One of the premises of the Banco de Olhos de Sorocaba-São Paulo is that everyone must be considered as a potential donor to overcome the general public’s fear of overlooked opportunities for donation 11. In addition, it argues that the most appropriate way to reach the public is through the media and to emphasize the positive aspect of organ and tissue donation: namely, saving lives 11.

Other examples of success in Brazil are the “Fundação Banco de Olhos de Goiás” and the “Banco de Olhos do Hospital das Clínicas da Faculdade de Medicina de Ribeirão Preto-São Paulo”. These organizations actively work with the population to increase the number of donations, to drastically reduce the waiting time and to popularize the concept of becoming a donor in these localities 11.

Spain, a global benchmark, has had a system of organ and tissue harvesting since 1990. This system, which is based on both a team training approach and use of the media, has increased the number of donations by 75% 12.

In Brazil, most potential organ and tissue donors are in intensive care units 13, which indicates the key role of intensivists 14, who generally appear to be in favor of the donation of organs and tissues but who rarely become actual donors 15.

Several authors agree that the organ and tissue shortage is due to a failure to convert potential donors into actual donors 16-21.

There are a number of problems in the donation process and in the process of obtaining organs and tissues for transplantation, including the health system’s lack of credibility, poor hospital infrastructure, a lack of information on transplants, an absence of additional benefits for medical teams, the reluctance of teams to interview families and a lack of medical training 22.

One study found that when an experienced physician interviewed families to obtain consent for the donation of an organ or tissue, an average of 80.3% of families agreed 23. When the interview was performed by an inexperienced physician, however, the rate of donation dropped to 35.5% 23.

Table 3 shows the reasons for refusal to donate organs and tissues per year in each Brazilian region. In every region, there were high rates of family refusal and medical contraindications.

This finding reflects Brazilian society’s ignorance about organ and tissue donation, which is caused by both a lack of information dissemination in the media and the unpreparedness of health professionals during their interviews with family members 18,24.

Importantly, families should be informed that removal of the cornea does not cause undesirable esthetic effects in the donor and that, in Brazil, this removal can only be performed after written permission has been obtained from the family 25.

The most positive changes have been observed in the Midwest region, especially in the state of Goiás, which now has the largest corneal transplant program in the country, and the Federal District, which has experienced the largest growth in the number of donors in the last two years, with a high proportion of potential donors reported as well 1.

States in the North region of Brazil, such as Pará, experience difficulty due to a high percentage of deaths from external causes and non-cooperation between municipalities and states, often motivated by political differences 2.

In the Northeast, two states appear to be having the most <1?A3B2 tlsb=-.02w?>success: Ceará and Pernambuco 1. These states have trained teams and organ and tissue transplant programs that have been established for more than 15 years and that are supported by the state governments, foundations and patient associations 1.

The Organ and Tissue Procurement Organization is authorized by the National Transplantation System and linked to the Center for the Notification, Procurement and Distribution of Organs. In particular, the organization supports and implements the identification, evaluation and feasibility assessment of potential organ and tissue donations within the limits defined by geographical and population criteria.

This initiative began in the state of São Paulo, but the following states now have units as well: Acre, Amazonas, Rondônia, Alagoas, Bahia, Ceará, Sergipe, Piauí, Rio Grande do Norte, Goiás, Mato Grosso do Sul, Minas Gerais, Rio de Janeiro, Rio Grande do Sul, Paraná and Santa Catarina. However, not all states have efficient programs for obtaining organs and tissues.

The states of Paraná and Santa Catarina have excellent transplant rates and high-quality continuing education programs for training teams and monitoring the performance of the Organ and Tissue Procurement Organization, in addition to supporting humanization and adopting host family-related donor strategies 2.

Although remarkable progress has been made in the last decade in every region in Brazil, the main obstacle to increasing the number of organ and tissue donations is physicians’ lack of preparation to approach family members upon the death of a potential donor.

Involving the media and improving the credibility of hospitals are mandatory for raising awareness and improving acceptance of organ and tissue donation in the population.

Thus, the present study urges the implementation of public policies that will allow all regions to more effectively meet the need for organ and tissue transplants, and especially corneal transplants. There are considerable disparities in the effectiveness and productivity of corneal transplant programs and the ability to perform corneal transplants among the regions of Brazil, with improvements in the South, Southeast and Midwest regions of Brazil and poor performance in the North and Northeast regions.

It is also important to emphasize that data are missing from the information provided by the RBT during the years evaluated. Moreover, there is no complete annual report from the Ministry of Health to provide statistics on organ and corneal transplants in Brazil. There is therefore a need to standardize the data made available to the public, health professionals, and the large collection of regulatory bodies to ensure the proper reporting of annual variables so that further research can be conducted on this topic in Brazil.

Furthermore, studies should be conducted in the Brazilian states with the worst donation performance, the lowest corneal transplant rates and the lowest rates of meeting transplant needs to help to identify the causes of the problem and to develop effective solutions.

## AUTHOR CONTRIBUTIONS

Almeida HG, Kara-Junior N and Hida RY made substantial contributions to the study conception and design, the acquisition, analysis and interpretation of the data, and the manuscript drafting.

## Appendix

Geographical map of Brazil.

## Figures and Tables

**Figure 3:**
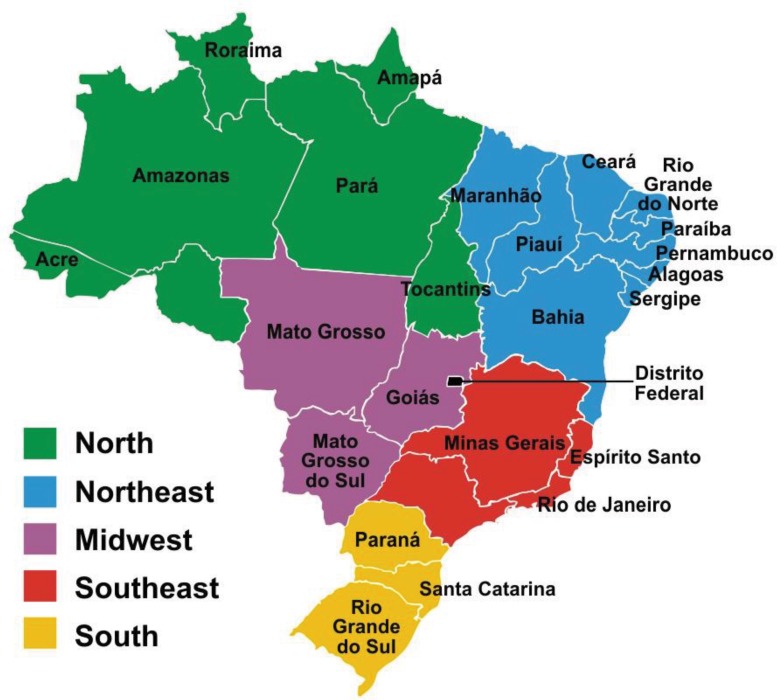


**Table 1:** Absolute numbers of individuals on the corneal transplant waiting list in each Brazilian region from 2012 to 2014.

REGION	ABSOLUTE NUMBER ON WAITING LIST		
	2012	2013	2014
North	979	961	1174
Northeast	2700	2400	3008
Midwest	778	168*	1065
Southeast	910	1385	3076
South	594	465	279

* In 2013, data from Goiás State were not available.

The data reflect the number of persons on the waiting list in December of each year.

Data for previous years were not available in the Brazilian Transplant Registry (RBT).

**Table 2:** Demand for corneal transplantation per year (DCT/Y) from 2012 to 2014 in each Brazilian region.

REGION	PERCENTAGE (%)		
	2012	2013	2014
North	33.0	31.3	27.5
Northeast	53.3	54.5	47.2
Midwest	70.0	89.1	55.7
Southeast	87.4	83.5	67.1
South	80.0	81.3	88.9

Data for previous years were not available in the Brazilian Transplant Registry (RBT).

**Table 3:** Reasons for refusal to donate organs in each Brazilian region from 2002 to 2014.

REGION	REASON FOR REFUSAL	YEAR												
		2002	2003	2004	2005	2006	2007	2008	2009	2010	2011	2012	2013	2014
**NORTH**	**NAF**	28	15	13	31	52	41	38	32	47	66	108	119	118
	**CIM**	60	69	58	65	78	39	25	55	21	23	52	87	50
	**BDNC**	0	0	1	3	6	0	7	21	6	18	52	33	22
	**II**	0	4	0	4	0	0	0	0	2	0	0	0	0
	**OTHER**	0	3	0	0	0	27	23	19	25	31	49	86	141
**NORTHEAST**	**NAF**	222	323	289	268	316	290	313	285	436	459	622	719	741
	**CIM**	138	157	302	271	406	336	195	217	220	208	280	329	408
	**BDNC**	15	3	13	24	123	130	262	266	279	321	353	154	156
	**II**	14	19	7	3	3	12	2	0	17	3	0	0	0
	**OTHER**	46	92	87	48	32	38	145	204	236	231	227	411	424
**MIDWEST**	**NAF**	157	161	133	102	115	108	91	94	129	100	119	192	221
	**CIM**	183	187	272	271	303	183	187	237	171	192	186	191	268
	**BDNC**	3	6	16	15	28	12	87	9	42	48	76	143	31
	**II**	7	1	11	5	29	9	5	2	1	4	0	0	0
	**OTHER**	21	39	29	11	38	108	67	91	129	173	192	139	172
**SOUTHEAST**	**NAF**	766	808	717	680	790	753	616	683	908	963	1029	1137	1059
	**CIM**	859	860	891	870	1055	990	288	374	325	247	188	281	336
	**BDNC**	55	37	34	24	17	26	32	45	46	74	211	715	682
	**II**	1	1	1	1	1	2	1	0	3	5	0	0	0
	**OTHER**	321	407	390	374	412	599	1668	1530	1340	1241	1016	579	755
**SOUTH**	**NAF**	112	143	207	254	266	315	271	296	280	349	437	455	471
	**CIM**	154	137	199	254	331	284	144	218	254	162	130	262	287
	**BDNC**	0	2	5	0	12	26	6	10	4	3	6	247	265
	**II**	2	2	17	29	29	0	1	0	0	22	0	0	0
	**OTHER**	40	58	57	29	76	16	206	144	160	247	286	66	31
**TOTAL**		3204	3534	3749	3636	4518	4344	4689	4832	5081	5190	5619	6345	6638

NAF: family refusal; CIM: medical contraindications; BDNC: brain death not confirmed; II: inadequate infrastructure.
